# High Accuracy and Miniature 2-D Wind Sensor for Boundary Layer Meteorological Observation †

**DOI:** 10.3390/s19051194

**Published:** 2019-03-08

**Authors:** Yichen Pan, Zhan Zhao, Rongjian Zhao, Zhen Fang, Hong Wu, Xianghua Niu, Lidong Du

**Affiliations:** 1 State Key Laboratory of Transducer Technology, Institute of Electronics, Chinese Academy of Sciences, Beijing 100190, China; panyichen.ucas@foxmail.com (Y.P.); rongjian8780@163.com (R.Z.); zfang@mail.ie.ac.cn (Z.F.); 2 School of Electrical, Electronic and Communication Engineering, University of Chinese Academy of Sciences, Beijing 100049, China; 3 Key Laboratory of Transportation Meteorology, China Meteorological Administration, Nanjing 210008, China; wu58283@163.com; 4 Beijing Institute of Applied Meteorology, Beijing 100029, China; nxh0322@126.com

**Keywords:** two-dimensional wind sensor, wind speed and direction, pressure difference, inclination correction, meteorological observation

## Abstract

Wind speed and direction are important parameters in meteorological observation. A solid wind sensor is needed with a small quadcopter for boundary layer meteorological observation. In this paper, the principle of a cylindrical two-dimensional wind sensor is reported and the data from wind tunnel experiments are analyzed. A model is proposed to describe the distribution of the pressure difference across a diameter of a cylinder, and the wind sensor is fabricated with MEMS (Micro-Electro-Mechanical System) differential pressure sensors. The wind sensor cylinder has a small size with a diameter of 30 mm and a height of 80 mm. In wind tunnel tests in the range of 1 to 40 m/s, the relative speed measuring errors and the direction measuring errors of the prototype are no more than ± (0.2 + 0.03 V) m/s (V is standard wind speed) and 5°, respectively. An inclination angle model is proposed to correct the influence of tilt angle on the quadcopter platform, the wind sensor can maintain the original wind speed and direction measurement accuracy within the 30° inclination range after compensation.

## 1. Introduction

Boundary layer meteorological observation is a developmental direction in the field of meteorological monitoring, which can determine various meteorological elements in the lower atmosphere and the vertical distribution of pollutants and their changes regularly. At present, the meteorological tower is still a kind of meteorological observation facility. The quadcopter has become increasingly common, capable, and affordable since its development, and their utility as a mobile sensor platform continues to grow [1,2].

Wind direction and speed are important parameters in meteorological observation. The traditional wind sensor is not suitable to apply to the quadcopter because of its large size and weight. Fortunately, MEMS technology pushed the development of the wind sensor and made devices with small size and low cost possible [3,4,5]. The thermal and mechanical sensitive elements of a wind sensor must be in direct contact with fluidic air which is easily polluted. The sensors can be damaged in a meteorological application [6]. In the process of meteorological observation with a wind sensor on the quadcopter, the swing of quadcopter has a great influence on the measurement. The inclination of the wind sensor has always been a hindrance to mobile measurement application, so the effects of inclination on meteorological parameters measurements will be discussed in this paper.

It is worth mentioning Bruschi P’s works in this paper, which is based on both ends of the diameter of the cylinder pressure difference to measure the two-dimensional velocity vector, design structure into the flow branch pipe to the pressure, and then use the principle of MEMS thermal flow sensor to measure the flow and calculate the wind speed and direction [7]. Massimo Piotto proposed a kind of cylindrical two-dimensional anemometer with two orthogonal measuring modules based on Bruschi P’s works [8]. Subsequently, he improved the design of the cylindrical two-dimensional anemometer by increasing the branch holes near each hole in the diameter and reducing the response curve error caused by different Reynolds numbers to 4% [2]. Similarly, Hao-Bing Liu proposed a cylindrical miniature two-dimensional anemometer to calculate wind speed vectors based on the pressure difference distribution on the cylindrical surface [9]. Literature [10] completed the prototype of cylindrical two-dimensional wind sensor, wind speed measurements, and standards of the anemometer and showed consistency with a wind measurement error of no more than 8°. However, all these wind sensors have some defects and cannot meet the need for boundary layer meteorological observation.

In this paper, the principle of the wind sensor is introduced based on the cylinder diametrical pressure difference. The mathematical model is established and based on a fabricated wind sensor in which four differential pressure sensors were used to measure the pressure instead of two flow sensors. It has the advantages of small size and high accuracy with no moving parts in the measurement of wind speed and direction and also has a higher protection ability gainst dust and rain compared with MEMS flow sensors. The relative speed measuring errors and the direction measuring errors of the prototype are no more than 5% and 5°, respectively. In addition, this paper studied the influence of the inclination angle of the wind sensor on the measurement results and established the inclination correction model and made inclination compensation of the wind sensor. After compensation, the wind sensor can maintain the original wind speed and direction measurement accuracy within the 30° inclination range.

## 2. Modeling

### 2.1. Modeling of Measurement

The research of cylindrical two-dimensional anemometer involves a fluid mechanics problem, that is the distribution of cylindrical surface pressure in the direction of vertical velocity in the flow field with uniform and constant velocity field. It is common to use pressure coefficient $C_{P}$ to describe cylindrical surface pressure distribution, and its definition is:

(1) $$C_{P}=\frac{P-P_{\infty }}{\frac{1}{2}\rho U_{\infty }^{2}}$$

**Figure 1:**
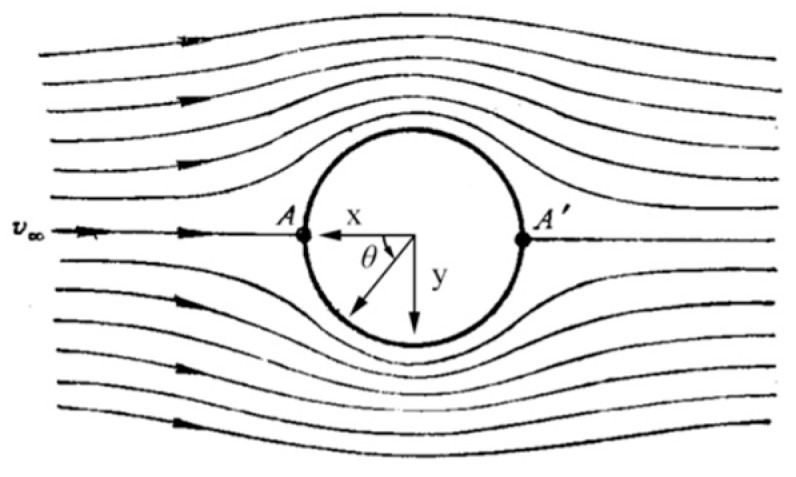
The ideal flow chart of cylindrical winding flow.

In which *p* is the object surface pressure, ρ, $P_{\infty }$ and $U_{\infty }$ are infinity fluid density, static pressure and flow rate, respectively. Figure 1 is the flow chart of the cylindrical winding under ideal conditions. Ideally, the pressure distribution of each section of an infinite length cylinder is consistent, and the pressure coefficient of a point on the cylindrical surface in this flow field is:

(2) $$C_{P}=1-4sin^{2}\theta$$

where θ is the azimuth angle of the point on the cylindrical surface in the coordinate system in Figure 1. 

Equation (2) shows that the pressures between the ends of a diameter in the column are equal. Point A and A′ are the stagnation point, at which the flow rate of the fluid is 0. Ideally, if the fluid viscosity is not considered, the resistance of the wind flow of any enclosed object is calculated to be 0. Actually, the fluid viscosity can cause the change of flow state so that the cylindrical surface pressure distribution has obvious differences to the ideal case. Using the fluid Reynolds number to determine the flow state, the definition is as follows [7]:

(3) Re=ρVDμ

where ρ, *V*, and *μ* are fluid density, velocity, and dynamic viscosity coefficient, respectively. *D* is the diameter of the cylinder. The Reynolds number reflects the ratio of the inertial force of the fluid to the viscous force, and the larger the Reynolds number, the smaller the relative inertial force of the viscous stress.

**Figure 2:**
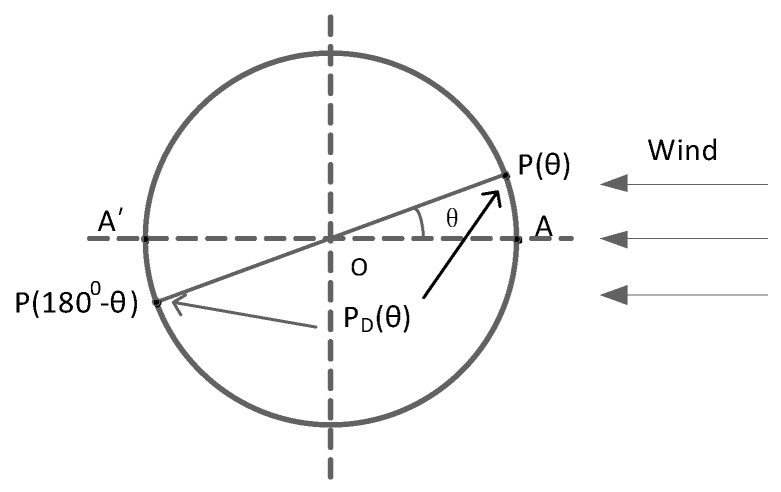
The pressure and pressure difference of a cylinder.

Figure 2 shows the pressure and pressure difference of a cylinder. P(θ) is the pressure of the point on the cylinder surface of which the diameter has an angle of θ between wind direction. There is a choked cylindrical pressure difference usually between the two ends of a diameter. Here $P_{D}\left(\theta \right)$ is defined as the pressure difference at both ends of the diameter. 

According to Equations (1) and (3), we can get the pressure difference between the point of the azimuth and the point at the other end of its diameter $P_{D}\left(\theta \right)$:

(4) $$P_{D}\left(\theta \right)=\frac{1}{2}\rho U_{\infty }^{2}\left[C_{P}\left(\theta \right)-C_{P}\left(180^{0}-\theta \right)\right]$$

According to Equation (1), the pressure differential coefficient of $C_{PD}\left(\theta \right)$ is defined as follows:

(5) $$C_{PD}\left(\theta \right)=\frac{P_{D}\left(\theta \right)}{\frac{1}{2}\rho U_{\infty }^{2}}$$

**Figure 3:**
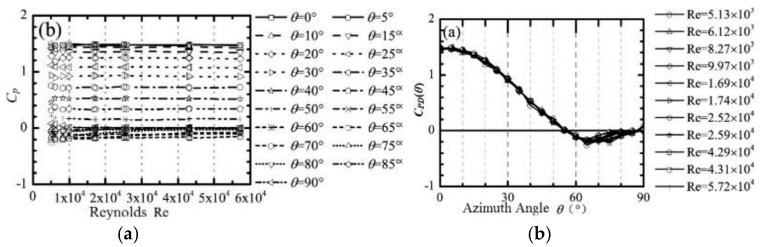
The measured pressure difference coefficients to (**a**) Reynolds numbers and (**b**) the azimuth angles.

**Figure 4:**
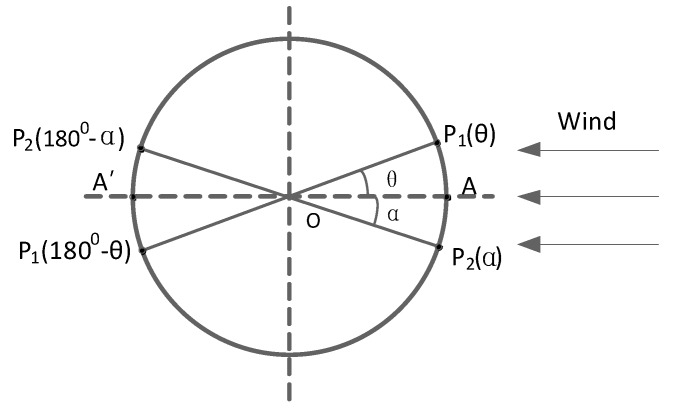
Distribution of $P_{1}$ and $P_{2}$.

Figure 3 shows the relationship of measured pressure difference coefficients with Reynolds numbers and azimuth angles. Figure 3a is a representation of the distribution of the pressure difference coefficient. Figure 3b shows the $C_{PD}\left(\theta \right)$ with non-monotonicity; it is monotonous about the azimuth distribution within the scope of 0° to 60°, but near 65° the value is extremum, so that the wind speed and direction need to be measured along four diameters in time in the range of 0° to 45°. Therefore, the mathematical model of the pressure difference on both ends is as follows:

(6) $$C_{PD}\left(\theta \right)=Acos^{2}\theta +B,\;\theta \in \left[0^{0},45^{0}\right]$$

where *A* and *B* are correction coefficients obtained by fitting the measured data. According to Equations (4)–(6), we can get the final expression formula of wind speed and wind direction.

(7) $$U_{\infty }=2\sqrt{\frac{P_{2}}{\rho \left(Asin2\theta +A+2B\right)}}$$

(8) $$\theta =\frac{1}{2}\left[arccos\frac{\left(\frac{P_{1}}{P_{2}}-1\right)\left(1+\frac{2B}{A}\right)}{\sqrt{{\left(\frac{P_{1}}{P_{2}}\right)}^{2}+1}}-arctan\frac{P_{1}}{P_{2}}\right]$$

where $P_{1}$, $P_{2}$ are the pressure value of the two diameter ends adjacent to each side of the front stagnation point as shown in Figure 4, and the angle of the diameter of these two points (θ+α) is 45°.

### 2.2. Modeling of Inclination Correction

**Figure 5:**
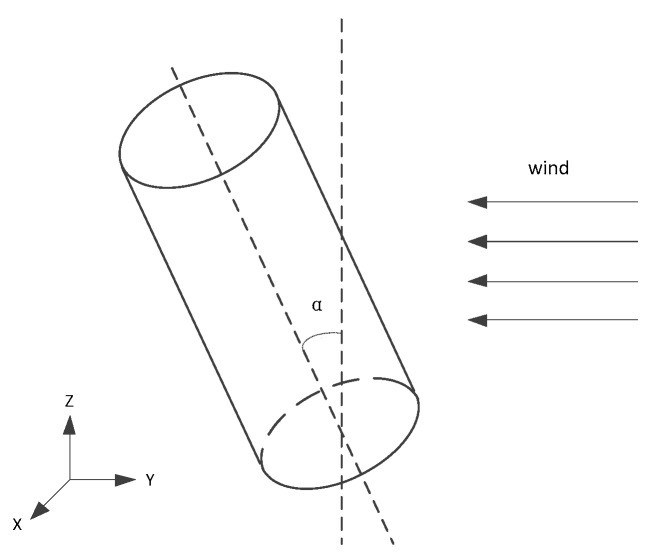
The definition of the inclination angle of the wind sensor.

In the modeling of the inclination, the wind was assumed to be two-dimensional wind, and there is no vertical component. Although it is not ruled out that the wind has a slight vertical component in the meteorological observation of the boundary layer, according to Weiyun Shao’s works, under the condition of Reynolds number Re=2.7×104, the inclination angle has no obvious effect on the measurement results within 15° [11]. In this paper, the inclination α was defined as the angle between the cylindrical axis and the vertical direction, as shown in Figure 5.

The hydrodynamic problem involved in the influence of inclination on wind speed measurement is the pressure distribution of finite length inclined cylinder. According to the principle of independence, the flow characteristics are processed only by the velocity component perpendicular to the cylindrical axis when the fluid tilts the cylinder around the flow [12]. According to this rule, when the inclination is α, the velocity component perpendicular to the direction of the cylindrical axis is:

(9) $$U_{C}=U_{\infty }\cdot cos\alpha$$

According to Equations (7) and (9), the wind speed at the inclination can be expressed as:

(10) $$U_{\infty }=\frac{2C}{cos\alpha }\sqrt{\frac{P_{2}}{\rho \left(Asin2\theta +A+2B\right)}}+D$$

where $U_{\infty }$ is the real wind speed when the wind sensor has an inclination; *C* and *D* are correction coefficients. Inclination has a little impact on wind direction measurements. $P_{1t}$ and $P_{2t}$ are the pressure difference when wind sensor tilts *α* degrees. According to the principle of independence, the relationship between the pressure difference $P_{t}$ and P can be approximately expressed as:

(11) $$P_{1t}=\frac{P_{1}}{cos\alpha }$$

(12) $$P_{2t}=\frac{P_{2}}{cos\alpha }$$

According to Equations (8), we find that when the wind sensor is tilted, the ratio of $P_{1}$ and $P_{2}$ is constant although the pressure difference changes. Therefore, the inclination has a little effect on the wind direction measurement. In this paper, only the influence of inclination angle on wind speed measurement will be discussed.

## 3. Prototype and Experiments

**Figure 6:**
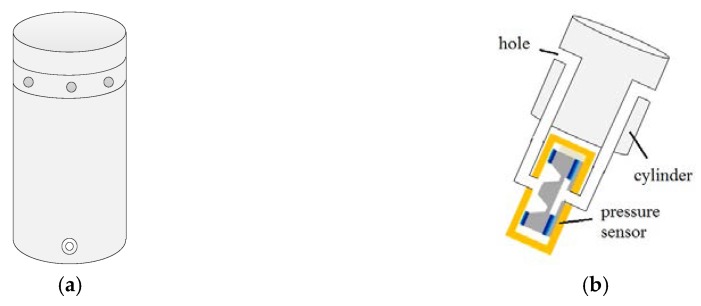
The prototype of the wind sensor. (**a**) Prototype of the wind sensor; (**b**) The principle of measurement.

Figure 6a shows the prototype of the wind sensor that consists of a 30 mm diameter, 80 mm cylindrical probe with four MEMS differential pressure sensor, a temperature sensor, MCU (Microcontroller Unit) and a peripheral circuit. The mass of the cylinder as a whole is 61 g, and the power of the wind sensor under working conditions is 210 mW. It has eight pressure holes with a diameter of 1 mm equally arranged in a cylindrical spacing between 45°. Pressure holes through the pipe are connected to the differential pressure sensors to measure the pressure difference on both ends of the diameter. Figure 6b shows the principle of measurement, which describes simply the connection between the differential pressure sensor and circular holes.

**Figure 7:**
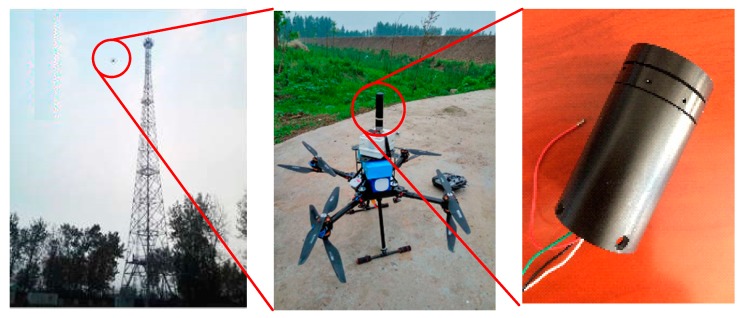
The quadcopter meteorological application.

Figure 7 shows the quadcopter meteorological application. The right image in Figure 7 shows the prototype of the wind sensor, which has a cylindrical metal shell. The middle image and left image in Figure 7 show the wind sensor application on a quadcopter and testing at the meteorological tower in Guandu Experimental Base in China.

**Figure 8:**
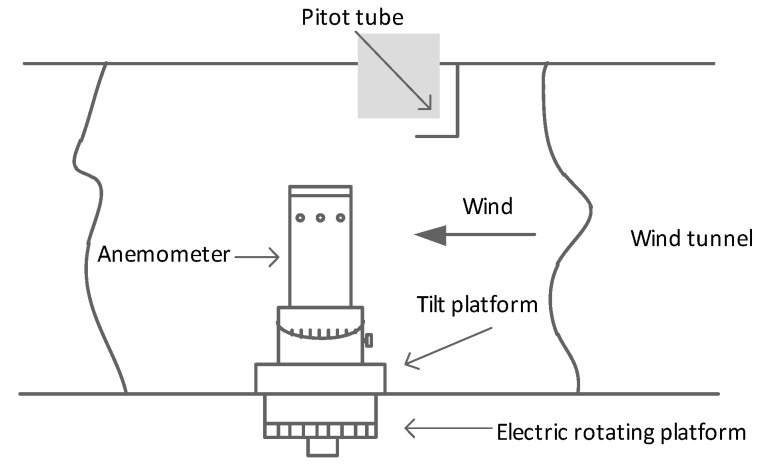
Standard wind tunnel for test prototype.

The prototype was calibrated in a standard wind tunnel, as shown in Figure 8. The wind tunnel has a wind speed range from 0.5m/s to 40m/s. The reference wind speed was detected by a Pitot tube, an air manometer, and a thermometer. The dynamic pressure was measured by a differential pressure gauge through the Pitot tube. To test wind direction, the prototype was rotated by an electric rotating platform with a range of 0° to 360° and the accuracy of 0.1°. The inclination experiment was tested by a tilt platform with a range of −45° to 45°and the accuracy of 1°. 

The experiments were performed in 360° angle range with a step of 10°. At each angle, the wind speed changes from 1m/s to 40m/s. To avoid the return impact of the test, the experiments were repeated in different wind speeds which increased from a low speed to high and decreased to low again. The measurement of the inclination was performed in ±30° tilt angle range with a step of 10° and the above measurement were repeated at each tilt angle.

## 4. Results and Discussion

When the sensor completed the calibration, the wind speed and direction were tested and compared with wind tunnel setpoints as standard data. According to the results, the measurement error of wind direction was no more than 5°, and the measurement error of wind speed was within ±(0.2 + 0.03 V) m/s, where V is the standard wind speed values. 

**Figure 9:**
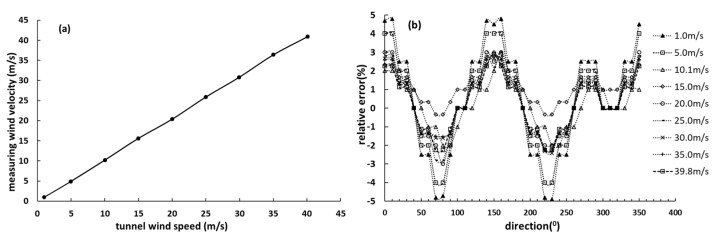
The wind speed test results, (**a**) linear wind speeds relationship and (**b**) the relative errors.

**Figure 10:**
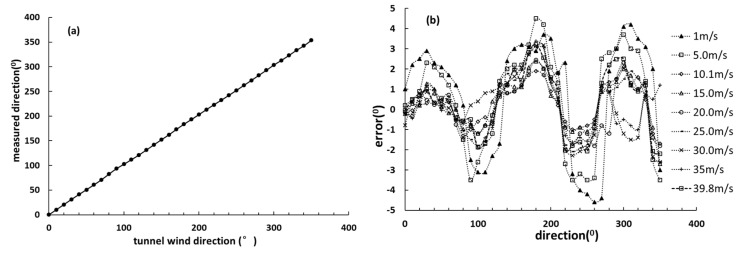
The wind direction test results, (**a**) The linear wind direction relationship and (**b**) the relative errors.

To represent the overall distribution of wind speed and direction measurement errors more intuitively, the relationship between the given tunnel wind speed and measured speed by the designed wind sensor for each wind speed are shown in Figure 9 and Figure 10. According to Figure 9 and Figure 10, the relative error of wind speed is not more than ±5%, and the wind direction error is not more than ±5°. As shown in Figure 9 and Figure 10, we can find that the relative error of wind speed and direction at 1 m/s and 5 m/s is larger obviously than others, the possible reason is that the measurement error of the differential pressure sensor at lower wind speed is greater than that of higher speed points. In addition, the Reynolds number is another important factor. In the measurement model, the Reynolds number is a fixed value, but $C_{PD}\left(\theta \right)$ curves are not coincident in all Reynolds numbers according to Figure 3b. Therefore, there must be an error when the Reynolds number is a fixed value. If the Reynolds number is dynamically changing, which means that the Reynolds number with the least error of the measurement result is selected according to the different wind speed, the wind sensor will perform better at low wind speed. 

In the actual measurement, we found that the wind speed error increases overall with the increase of standard values because of the measurement error of differential pressure sensor is magnified linearly with the increase of wind speed, but it is still within the expected range. The wind direction error has the trend overall of high, if the measurement data is further fitted and compensated, the measured result could be better.

The wind sensor was tested at different inclination angles within the range of 0° to 30° without the inclination correction model, and the wind speed measurement errors vary greatly under different inclination angles. The maximum error is 20%, which is a lot bigger than 5%. After combining the data with the model and doing regression analysis, the values of compensation coefficients *C* and *D* at different inclination angles were obtained, which are listed in Table 1. It is clear that coefficients *C* and *D* are not the same but similar at different angles, so we use the arithmetic averages of *C* and *D* as the final correction coefficient.

(13) $$C=\bar{C}=1.039$$

(14) $$D=\bar{D}=0.48$$

**Figure 11:**
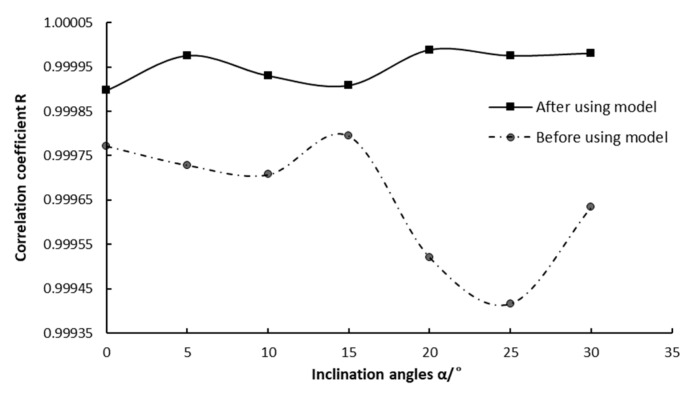
Comparison of wind speed correlation coefficient before and after using the inclination correction model.

The wind sensor was tested using the inclination correction model with the obtained coefficient, and the results are shown in Figure 11. It is intuitive to see a very significant improvement in the correlation coefficient after using the inclination correction model. According to the measured results, the wind speed measurement error is within 5% of the inclination range of 0° to 30°.

**Figure 12:**
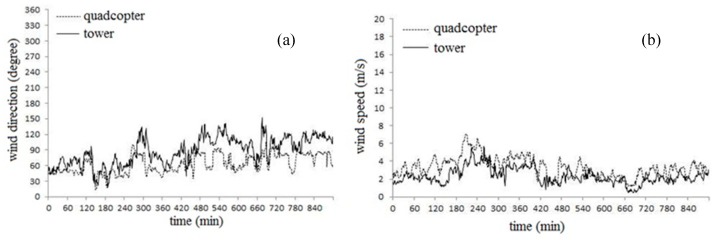
Boundary layer meteorological observation test compared in the height 70 m between our wind sensor on a quadcopter and traditional wind sensor in a tower, (**a**) wind direction and (**b**) wind speed.

We have tested the wind sensor in a quadcopter and compared with cup wind sensor in the tower, and the results are shown in Figure 12. The wind speed and direction measurements of the wind sensor followed the dynamic changes of the wind cup sensor. The reason why the wind speed and wind direction measurement values are not exactly the same is that the wind speed is relatively small, and the measurement error of wind sensor is relatively large at low wind speed. If the wind speed increases, the test result would be better. In general, the wind sensor can meet the needs of meteorological observation at the boundary layer.

## 5. Conclusions

In this paper, a two-dimensional wind sensor measuring principle was proposed. A mathematical model was established to describe the choke pressure difference distribution on both ends of the cylinder diameter. According to the principle, a prototype of a two-dimensional wind sensor based on MEMS differential pressure sensor was fabricated, which has the properties of small size and high accuracy. Prototypes have been used with the quadcopter for boundary layer meteorological observation application. The test results of the two-dimensional wind sensor indicate that the wind speed and wind direction measurement error are no more than 5% and 5° respectively, which meets the needs of meteorological observation at the boundary layer.

In addition, the effect of inclination on wind speed measurement of the wind sensor was studied in this paper. An inclination correction model was established to describe the pressure distribution of finite length inclined cylinder on the surface in flow field. The test results indicate that the mathematical model can correct the effect of the inclination angle to the test so that the wind sensor can maintain the original measurement accuracy in the inclination range of 0° to 30°.

Although the wind sensor prototype performed well, on the whole, the error at low wind speed points is still a bit large, which is a problem worthy of further study and exploration. In this paper, the Reynolds number was a definite value, and changing the Reynolds number dynamically with the wind speed may be the breakthrough to solve the problem.

## Figures and Tables

**Table 1:** Coefficients table under different inclination angles.

α	1/cosα	*C*	*D*
0	1.000	1.001	0.51
5	0.996	1.029	0.46
10	0.985	1.038	0.48
15	0.966	1.079	0.43
20	0.940	1.074	0.41
25	0.906	1.046	0.52
30	0.866	1.008	0.52
